# Effects of Mining Activities on *Gerbillus nanus* in Saudi Arabia: A Biochemical and Histological Study

**DOI:** 10.3390/ani9090664

**Published:** 2019-09-07

**Authors:** Ahmed M. Almalki, Jamaan Ajarem, Naif Altoom, Fahed S. Al-Otaibi, Saleh N. Maodaa, Ahmed A. Allam, Ayman M. Mahmoud

**Affiliations:** 1 Zoology Department, College of Science, King Saud University, Riyadh 11451, Saudi Arabia (A.M.A.) (F.S.A.-O.) (S.N.M.); 2 Department of Biology, King Khalid Military Academy, Riyadh 14625, Saudi Arabia; 3 Zoology Department, Faculty of Science, Beni-Suef University, Beni-Suef 62514, Egypt

**Keywords:** heavy metals, mining, Balochistan gerbil, liver injury, kidney damage

## Abstract

**Simple Summary:**

Mining is the extraction of materials of economic importance from the earth. It is an important source of employment and economic development. However, mining activities can impact on biodiversity, the environment, and human health, mainly through the emission of large quantities of heavy metals. Our objective was to investigate the impacts of gold mining on the Balochistan gerbil, a rodent that inhabits the natural environments of Saudi Arabia. Our results demonstrate the accumulation of heavy metals in the soil, in Arabian boxthorn plants, and in the different tissues of the gerbils. In addition, the gerbils exhibited liver, kidney, and lung injury associated with decreased antioxidants. These data may be of public interest and may call attention to the evaluation of the impacts of gold mining on the environment and nearby communities.

**Abstract:**

Mining can impact the environment, biodiversity, and human health through direct and indirect practices. This study investigated the effects of gold mining on *Gerbillus nanus*, in relation to organ dysfunction and redox imbalance. Soil samples, *Lycium shawii*, and *G. nanus* were collected from a site near a mining plant, and a control site. Soil and *L. shawii* samples from the mining site showed significantly higher cadmium (Cd), copper (Cu), mercury (Hg), arsenic (As), zinc (Zn), lead (Pb), and vanadium (V) levels. Hepatic, renal, and pulmonary Cd, Pb, Hg, Zn, Cu, Fe, As, and V concentrations were significantly higher in *G. nanus* from the mining site. Markers of liver and kidney function were elevated in serum, and several histological manifestations were observed in the liver, kidney, and lung of *G. nanus* from the mining site. Malondialdehyde and nitric oxide levels increased, and glutathione and antioxidant enzymes decreased in the liver and kidney of *G. nanus*. In conclusion, mining practices trigger tissue damage and oxidative stress in *G. nanus* that live close to the mining site. These findings can represent a scientific basis for evaluating the environmental and health impacts of mining on nearby communities.

## 1. Introduction

Mining refers to the extraction of geological materials such as minerals, coal, limestone, and other materials of economic interest from the earth. Mining activities include both small- and large-scale activities, and have a significant contribution to economic growth and development. However, mining activities can impact the environmental and social systems, living standards, health, and traditional practices of nearby communities [1,2,3]. Erosion, altered soil profile, contamination of soil and local streams, and emissions are among the environmental impacts that result from the construction, operation, and maintenance of mines [4,5]. Despite its importance as a source of employment, and the progress in occupational health and safety, the mining sector remains a high-risk environment where workers are highly susceptible to occupational injuries [6].

The widespread contamination of the environment with heavy metals (HMs) is one of the main consequences of mining [7]. Owing to their non-degradative nature, an increase in HM concentrations can exert long-term negative impacts on the ecosystem and human health [8]. HMs possess a high tendency for bioaccumulation and biomagnification; hence, they trigger serious health problems [9,10]. Additionally, several reports have investigated the toxic in vitro and in vivo effects of HMs [11,12]. Lead (Pb), cadmium (Cd), arsenic (As), mercury (Hg), zinc (Zn), and copper (Cu) have been identified in mining waste. Some of these HMs function as essential micronutrients and play central roles in redox processes, biochemical reactions, and the electron transport chain, whereas the nonessential HMs have no biological importance and are very toxic to living organisms [13]. Cd is a potent HM pollutant with a high tendency for bioaccumulation in living organisms [14]. Exposure to Cd is common in the mining industry, and hence poses a health threat to humans [15]. Pb is a well-documented HM pollutant with hazardous health effects and a high worldwide emission rate [9,14]. The long-term exposure to Pb and Cd is linked to the development of liver and neurological disorders, cancer, osteoporosis [11,16], and cardiovascular disease [17]. In rats chronically exposed to Pb and Cd, liver and heart tissue injuries and oxidative damage have been observed [12]. As is a very dangerous pollutant that attracts significant worldwide attention due to its potential toxic effects [18]. Lung, kidney, and skin cancers, cardiovascular and neural alterations, hepatomegaly, peripheral vascular disease, and other diseases may be triggered from exposure to As (reviewed in [19]). Hg is another HM that can induce cell death by binding the cysteine residues of proteins and depleting cellular antioxidants [20].

Given the hazardous impact of mining, and the resulting HM waste on the environment, society, and health, we investigated the effect of mining on the Balochistan gerbil (*Gerbillus nanus*) in Riyadh (Saudi Arabia). *G. nanus* belongs to the murid subfamily Gerbillinae [21] and inhabits natural environments in Saudi Arabia [22], and is therefore selected for investigation.

## 2. Materials and Methods

### 2.1. Study Site and Collection of Samples

Samples were collected from the area of mining activities in Al-Quway’iyah, a city located 165 km west of Riyadh Province (Saudi Arabia). This city is one of the large governorates in Saudi Arabia, and hence selected for the study.

**Figure 1:**
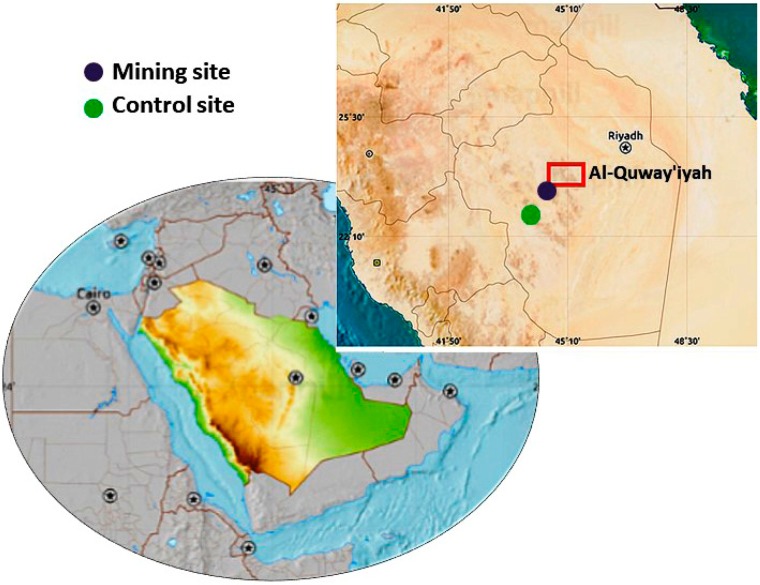
Location map of the mining and control sites.

The investigated gold mine site is located southwest of Al-Quway’iyah city. The samples were collected from two sites, as follows (Figure 1):

Site 1 (Mining): Located 500 m away from the gold mine, between E45° 05′ and N23° 47′.
Site 2 (Control): Located 20,000 m away from the gold mine, between E45° 06′ and N23° 36′.

Samples of the soil at 20 cm depth (*n* = 8) and *Lycium shawii* (Arabian boxthorn) (*n* = 8) were collected from both sites for the determination of HMs. Eight *G. nanus* were collected from each site with the help of specific rodent traps and transferred to the laboratory. The animals were sacrificed under anesthesia, and blood and tissue samples were collected for analysis. The protocol and procedures were approved by the institutional animal ethics committee of King Saud University (No. KSU-20165).

### 2.2. Determination of HMs

The concentrations of HMs were determined in the soil, *L. shawii*, and *G. nanus* kidneys, liver, and lungs using ELAN 9000 ICP/MS (Perkin Elmer Sciex Instruments, Concord, ON, Canada).

### 2.3. Assay of Liver and Kidney Function Markers

Serum alanine transaminase (ALT), aspartate transaminase (AST), alkaline phosphatase (ALP), creatinine, urea, and uric acid were measured using Biomerieux (Craponne, France) reagent kits, following the manufacturer’s instructions.

### 2.4. Assay of Oxidative Stress Markers and Antioxidants

Samples from the liver and kidney were homogenized in phosphate buffered saline (10% w/v) and centrifuged, and the supernatant was used for the assays. Malondialdehyde (MDA) [23], nitric oxide (NO) [24], reduced glutathione (GSH) [25], superoxide dismutase (SOD) [26], and catalase (CAT) [27] were assayed in the liver and kidney homogenate samples. Protein content was determined using Bradford assay.

### 2.5. Histological Study

Samples from the kidneys, liver, and lungs of *G. nanus* were fixed with 10% neutral buffered formalin and processed for paraffin embedding. 5 μm sections were cut, stained with hematoxylin and eosin (H&E), and examined using a light microscope.

### 2.6. Statistical Analysis

Data were analyzed using GraphPad Prism 7 (La Jolla, CA, USA) and expressed as means ± standard error of means (SEM). All statistical comparisons were performed using *t*-test, and a *p* value <0.05 was considered significant.

## 3. Results

### 3.1. HM Concentration in Soil and L. shawii Samples

**Figure 2:**
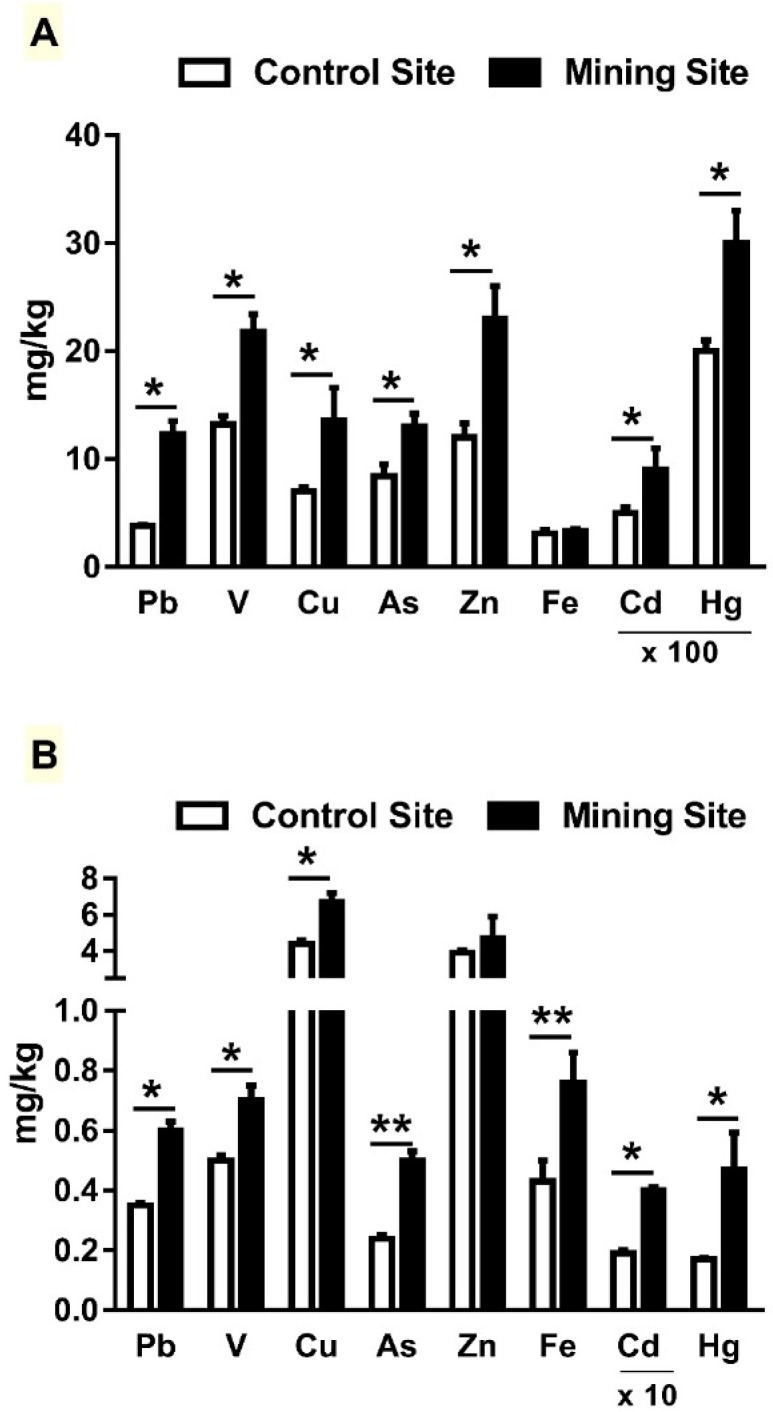
Concentration of heavy metals in (**A**) soil and (**B**) *L. shawii* from the mining and control sites. Data are means ± SEM, (*n* = 8). * *p* < 0.05 and ** *p* < 0.01.

The concentration of HMs has been determined in soil samples collected from the control and mining sites. Pb, V, Cu, As, Zn, Cd, and Hg concentrations were significantly higher (*p* < 0.05) at the mining site, when compared with the control site (Figure 2A). The results revealed non-significant difference in Fe concentration between both sites. *L. shawii* samples at the mining site showed higher concentrations of Pb, V, Cu, As, Fe, Cd, and Hg, whereas Zn concentration was not significantly different when compared with the control site (Figure 2B).

### 3.2. Concentration of HMs in Liver, Kidneys and Lungs of G. nanus

**Figure 3:**
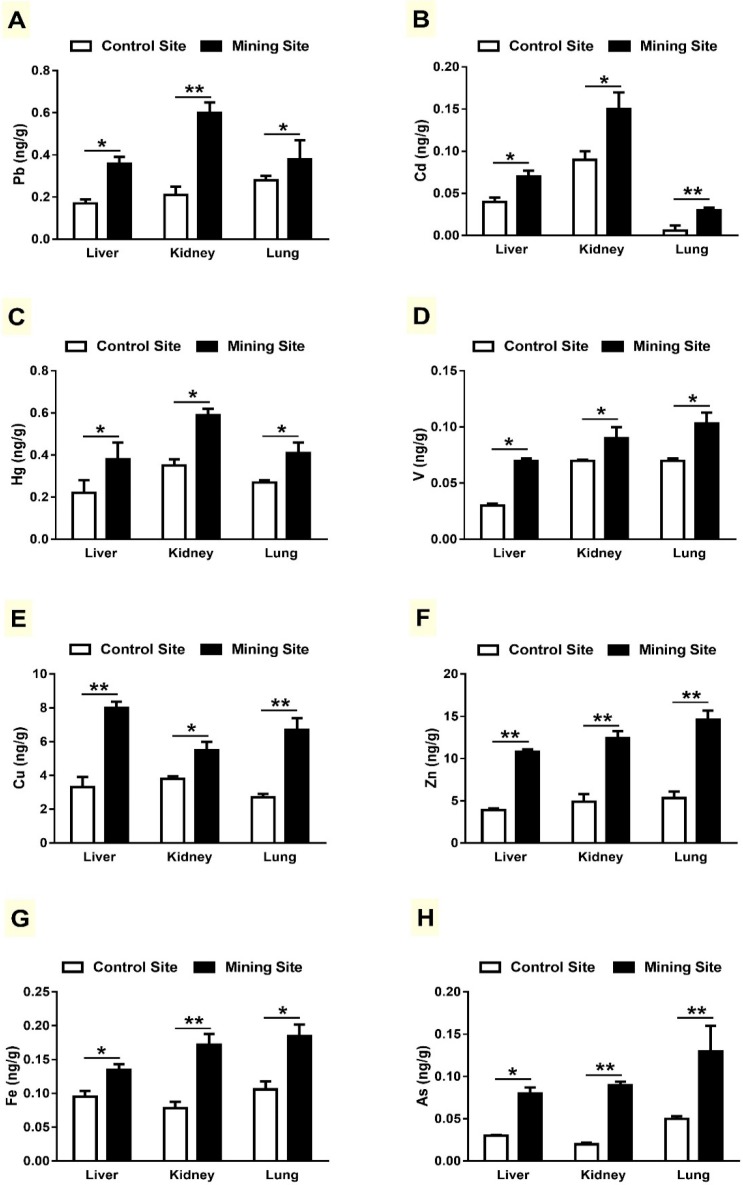
Concentration of heavy metals in the liver, kidneys and lungs of *G. nanus* from the mining and control sites. Data are means ± SEM, (*n* = 8). * *p* < 0.05 and ** *p* < 0.01.

Pb, Cd, and Hg levels were significantly higher in the liver, kidneys, and lungs of *G. nanus* collected from the mining site, when compared with the control site animals, as depicted in Figure 3A–C. Similarly, concentrations of V (Figure 3D), Cu (Figure 3E), and Zn (Figure 3F) were significantly higher in the target tissues of *G. nanus* from the mining site. Concentrations of Fe (Figure 3G) and As (Figure 3H) were also remarkably higher in the liver, kidneys, and lungs of *G. nanus* from the mining site.

### 3.3. Effect of Mining on Liver Function and Histology in G. nanus

**Figure 4:**
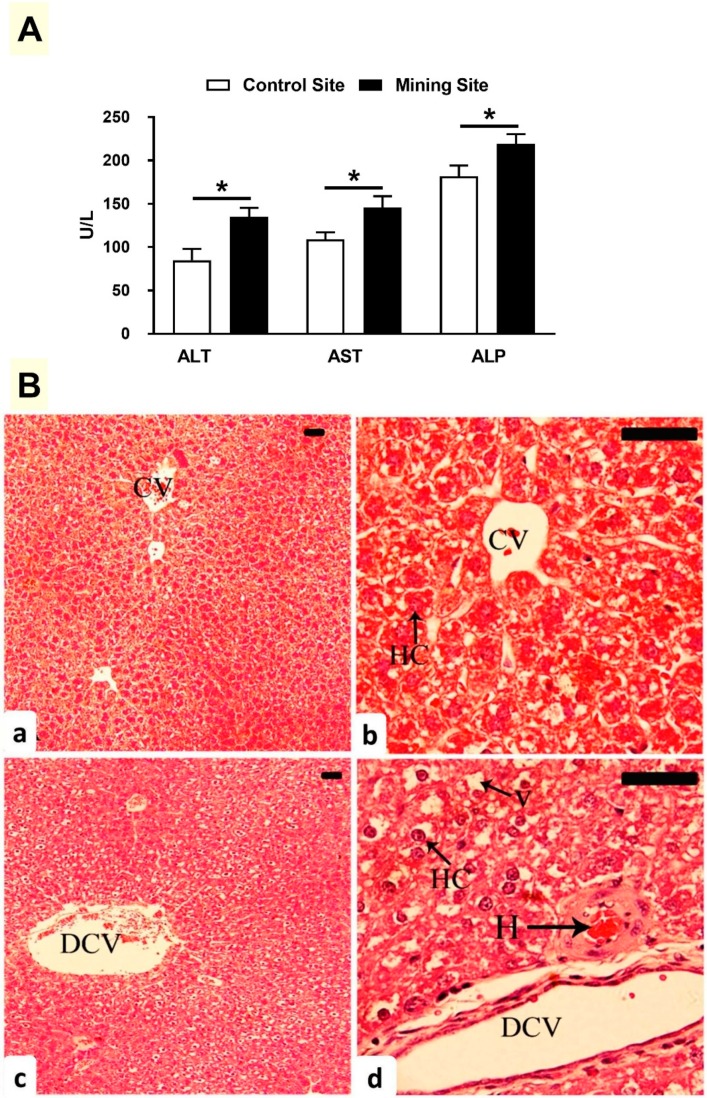
(**A**) Effect of mining activities on liver function markers of *G. nanus*. Data are means ± SEM, (*n* = 8). * *p* < 0.05. (**B**) Photomicrographs of H&E-stained sections in the liver of *G. nanus* from the control site (**a**,**b**) showing normal structure of hepatocytes (HC) and central vein (CV); and animals from the mining site (**c**,**d**) showing a dilated central vein (DCV), hemorrhage (H), and vacuolations (V). Scale bar = 100 µm.

The liver function markers ALT, AST, and ALP were elevated in the serum of *G. nanus* from the mining site (*p* < 0.05) when compared with the corresponding control site animals (Figure 4A). Examination of the liver section showed normal structure of the hepatic lobules and hepatocytes in *G. nauns* from the control site (Figure 4B(a,b)). By contrast, *G. nanus* from the mining site showed hepatocyte vacuolations, hemorrhage, and dilated central veins (Figure 4B(c,d)).

### 3.4. Effect of Mining on Kidney Function and Histology in G. nanus

**Figure 5:**
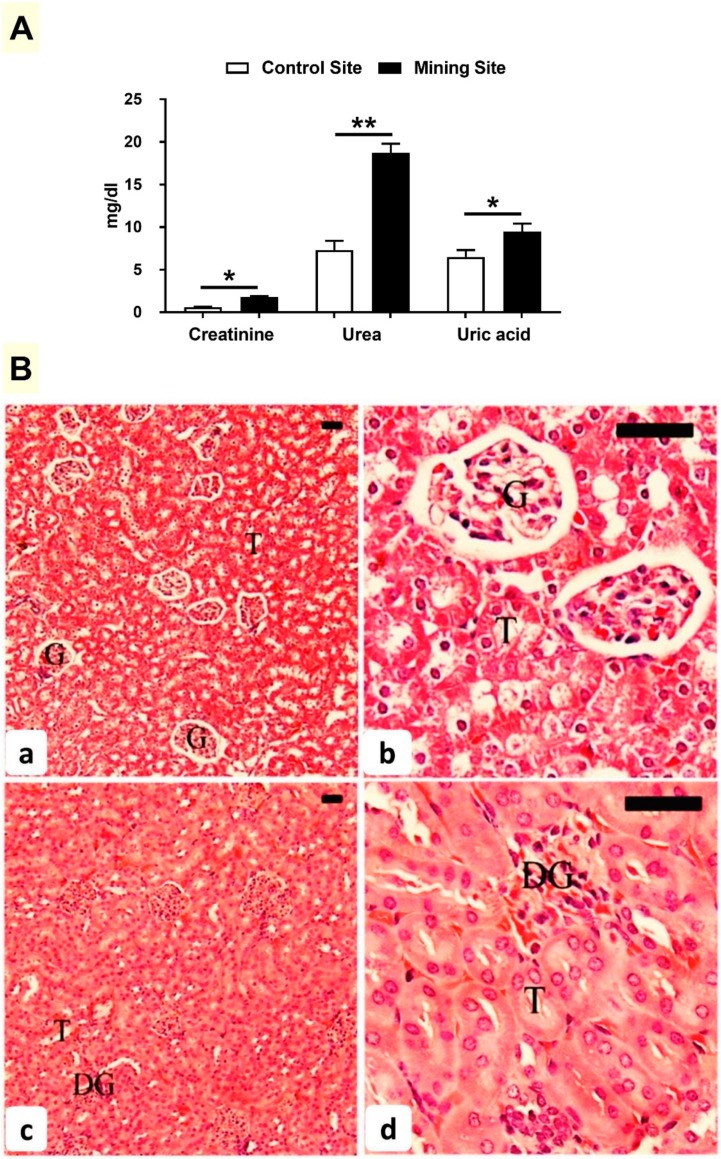
(**A**) Effect of mining activities on renal function markers of *G. nanus*. Data are means ± SEM, (*n* = 8). * *p* < 0.05 and ** *p* < 0.01. (**B**) Photomicrographs of H&E-stained sections in the kidney of *G. nanus* from the control site (**a**,**b**) showing normal structure of the glomeruli (G) and renal tubules (T); and animals from the mining site (**c**,**d**) showing degenerated glomeruli (DG). Scale bar = 100 µm.

Serum creatinine, urea, and uric acid were elevated significantly (*p* < 0.05; *p* < 0.01; *p* < 0.05) in *G. nanus* from the mining site (Figure 5A). While *G. nanus* from the control site showed normal kidney (Figure 5B(a,b)), animals from the mining site exhibited glomerular degeneration (Figure 5B(c,d)).

### 3.5. Effect of Mining on the Lung of G. nanus

**Figure 6:**
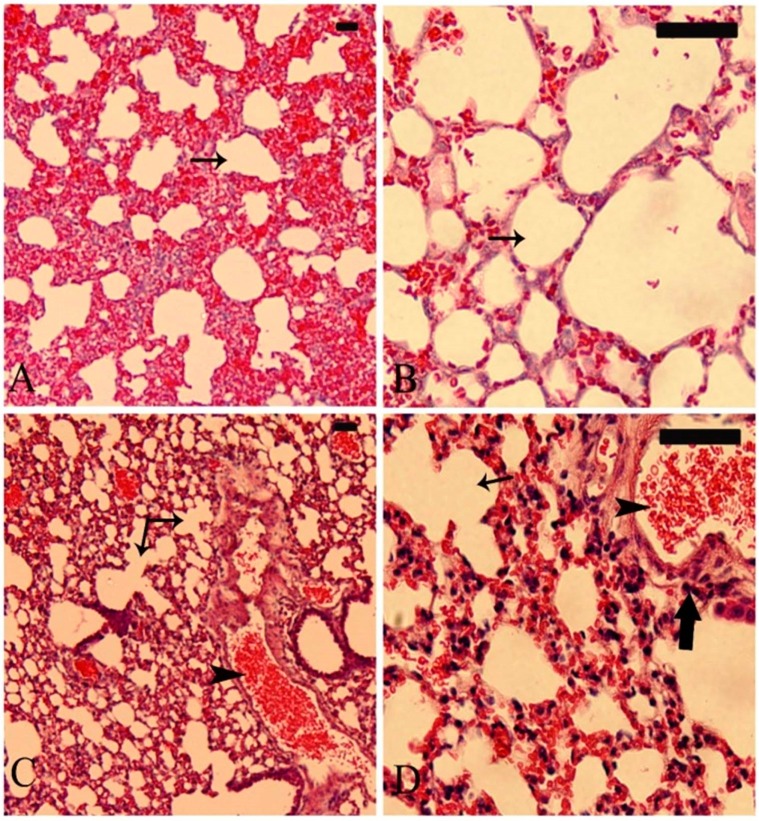
Photomicrographs of H&E-stained sections in lung sample of *G. nanus* from the control site (**A**,**B**) showing normal structure of the bronchioles and alveoli (arrow), and the lung of animals from the mining site (**C**,**D**) showing dilated alveoli (thin arrow), inflammatory cell infiltration (thick arrow), and congested blood vessels (arrow head). Scale bar = 100 µm.

**Figure 7:**
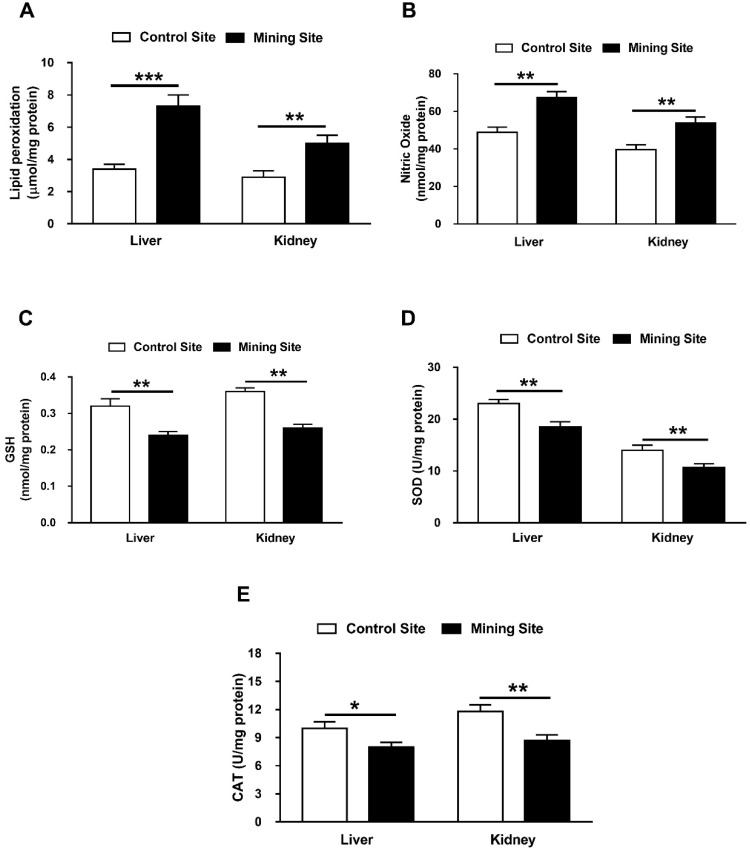
Effect of mining activities on oxidative stress and antioxidants in liver and kidney of *G. nanus*. Animals from the mining site showed an increase in (**A**) lipid peroxidation and (**B**) nitric oxide, and decreased (**C**) GSH, (**D**) SOD, and (**E**) CAT in liver and kidney. Data are means ± SEM, (*n* = 8). * *p* < 0.05, ** *p* < 0.01 and *** *p* < 0.001.

As represented in Figure 6A,B, the lung of *G. nanus* from the control site showed normal structure of the bronchioles and alveoli. By contrast, the lungs of animals from the mining site showed dilated alveoli, inflammatory cell infiltration, and congested blood vessels (Figure 7C,D).

### 3.6. Effect of Mining on Hepatic and Renal Redox Balance in G. nanus

To evaluate the impact of mining on the redox balance in *G. nanus*, we determined lipid peroxidation (LPO), NO, and cellular antioxidants in the liver and kidney. *G. nanus* from the mining site showed a remarkable increase of hepatic (*p* < 0.001) and renal (*p* < 0.01) LPO (Figure 7A). Similarly, hepatic, and renal NO was increased significantly (*p* < 0.01) in *G. nanus* from the mining site (Figure 7B). When compared with the control site, animals from the mining site showed a decrease in hepatic and renal GSH content (*p* < 0.01; *p* < 0.01; Figure 7C), SOD (*p* < 0.01; *p* < 0.01; Figure 7D), and CAT (*p* < 0.05; *p* < 0.01; Figure 7E).

## 4. Discussion

This study evaluated the impact of mining activities in Al-Quway’iyah, Riyadh (Saudi Arabia) on *G. nanus*, with an emphasis on HM accumulation and redox imbalance. To evaluate the emission and distribution of HMs at the mining site, we determined the concentrations of Pb, V, Cu, As, Zn, Cd, Fe, and Hg in soil, *L. shawii* samples, and different organs of *G. nanus*. Pb, V, Cu, As, Cd, and Hg were increased in both the collected samples of soil and *L. shawii*. Fe and Zn concentrations were not significantly increased in soil and *L. shawii*, respectively. These findings show the effect of mining on the soil profile, as well as on plants growing in the areas nearby.

Despite their negative impact on the ecosystem and adverse health effects, exposure to HMs continues to increase in many regions [28]. Here, Pb, V, Cu, As, Zn, Cd, Fe, and Hg concentrations were increased in the liver, kidneys, and lungs of *G. nanus*. Besides their environmental impact, these HMs have proven to be a major threat to the body and its proper functioning [28]. Accordingly, liver and kidney dysfunction, along with histopathological manifestations in the livers, kidneys, and lungs of *G. nanus* were observed. These hazardous effects are directly connected to the accumulation of HMs in different tissues of *G. nanus*.

Pb, Cd, As, and Hg were demonstrated to be common HM pollutants produced through various human activities, including mining, and are all hazardous to human health. Pb is a highly toxic HM whose widespread use has resulted in health problems as well as environmental contamination. It has been estimated that 540,000 deaths per year, particularly in developing countries, occur due to Pb contamination [29] and that 26 million people are at risk [30]. The most common depositories of Pb within the body are the liver and kidneys [31], and this very likely explains the observed hepato- and nephrotoxicity in *G. nanus* from the mining site. The accumulation of Pb in the liver and kidney elevated transaminases, ALP, and renal function markers, along with the histopathological changes indicated by the injury of liver and kidney tissues. In this context, *in vivo* studies have demonstrated both hepato- and nephrotoxicity in rodents exposed to low concentrations of Pb [32,33,34]. The toxicity of Pb has been attributed mainly to its ionic properties and induction of redox imbalance [35]. It can disturb cellular signaling and metabolism, ionic transportation, and enzyme activities by replacing mono- and bivalent cations [36]. In addition to its ionic activity, Pb triggers the generation of reactive oxygen species (ROS), which promotes oxidative damage to lipids, DNA, and proteins [37]. Accordingly, elevated LPO and NO was accompanied by a depletion of cellular antioxidants within the liver and kidneys of *G. nanus*, demonstrating an oxidative stress status. Furthermore, Pb concentration was increased in the lungs of *G. nanus*, which showed dilation of alveoli, infiltration of inflammatory cells, and congestions. The relationship between lung injury and Pb accumulation was supported by the findings of Li et al., who demonstrated increased Pb concentration in the lungs of rats following exposure to atmospheric fine particulate matter [38].

Cd is a highly toxic, nonessential HM and a serious environmental pollutant. When released into the environment, Cd can remain for several decades in the soil and sediment because of its lack of degradability [39,40]. It has a high rate of soil-to-plant transfer and therefore accumulates in plants, predominantly in vegetables and fruits, and ultimately reaches the human body [41]. Our study shows the accumulation of Cd in the soil and *L. shawii* samples from the mining site. In addition, Cd concentrations were increased in different tissues of *G. nanus*, and its accumulation was associated with oxidative stress, hepato-/nephrotoxicity, and lung injury. Although the exact mechanisms of Cd toxicity have not been fully elucidated, the role of oxidative stress has been well-acknowledged. Cd produces hydrogen peroxide, which acts as a source of free radicals generated via Fenton reaction [42]. Its toxicity has been reported to cause damage to the liver and kidneys [43,44]. Upon exposure, Cd binds to albumin and is taken up and deposits primarily in the liver [45]. Within the liver, Cd promotes ROS generation, LPO, membrane damage, GSH depletion, and cell death [46]. Cd binds to metallothionine, and the produced complexes induce liver injury and circulate to the kidney, where they accumulate and cause renal tissue injury [47]. The activity of SOD and CAT was decreased in the liver and kidney of *G. nanus* from the mining site, and was a result of the binding of Cd with the thiol groups of these enzymes [48]. In addition, Cd can inhibit complex III of the mitochondrial electron transport chain [48]. The observed nephrotoxicity in this study was associated with glomerular degeneration, which has been shown in rats exposed to Cd [49]. Furthermore, the lungs represent one of the main routes through which Cd enters the body [40]. In this study, *G. nanus* showed lung injury associated with elevated Cd concentrations. The potential of Cd to provoke pulmonary toxicity has been supported by studies reporting bronchial and pulmonary irritation, transient bronchial inflammation [50], diminished pulmonary function [51], and primary lung cancers [52] following exposure to Cd.

Mining is one of the major sources of As and Hg pollution. Accordingly, As and Hg were found in high concentrations in the soil, *L. shawii*, and different tissues of *G. nanus* collected from the mining site. As is extensively available in different forms and possesses toxic and carcinogenic effects [53]. The toxicity of As forms inorganic arsenic species (iAs), monomethylarsonic acid, and dimethylarsinic acid, which produces methylated arsenicals via enzymatic conversion. Monomethylarsonic acid, an intermediate product produced during the transformation of iAs, is highly toxic and carcinogenic [53]. The toxicity of As has been associated with its ability to increase ROS levels, activate apoptosis signaling, alter metabolism and energy generation, and replace metal ions in cellular biomolecules [54]. Therefore, the accumulation of As in *G. nanus* has a role in the observed oxidative stress and hepato-/nephrotoxicity. Recent reports have shown As-induced hepatic, renal, and pulmonary manifestations in experimental animals [55,56,57].

Hg is a well-known HM pollutant, and its toxicity is commonly responsible for acute HM poisoning. Although neurotoxicity is the most common effect of Hg and methylmercury, which induces LPO and cell death [58], it can also cause nephrotoxicity [59] and hepatotoxicity [60]. Hence, Hg accumulation in *G. nanus* contributes, at least in part, to liver and kidney injury and redox imbalance.

V, Cu, Zn, and Fe were the other HMs shown to accumulate in the soil, *L. shawii*, and different tissues of *G. nanus* from the mining site. V is an environmental pollutant that possesses hazardous effects, and the inhalation of vanadium pentoxide dust has caused occupational toxicity [61]. Liver injury and other toxic effects have been demonstrated in rats exposed to V [62], and inhalation of V caused bronchopneumonia and chronic productive cough [63,64]. Cu exists in oxidized Cu(II) and reduced Cu(I) states, and is therefore considered potentially toxic. Generation of ROS during the conversion between the oxidized and reduced states represents the main reason of Cu toxicity [65]. Zn is an essential micronutrient needed for vital cell functions in microorganisms, plants, and humans; however, concentrations beyond the physiological value are toxic. At high concentrations, Zn interacts with sulfhydryl groups or replaces other essential metals in different cellular proteins [66]. Iron is a vital nutrient that acts as a cofactor for multiple enzymes within living cells. It mediates reactions that can catalyze the formation of free radicals, leading to cell damage. Hence, iron overload can induce toxicity particularly in children [67], as well as hepatotoxicity [68] and nephrotoxicity [69]. Therefore, excess V, Cu, Zn, and Fe are associated with ROS generation and contribute to liver, kidney, and lung injury in *G. nanus* that live close to the mining site.

## 5. Conclusions

These findings highlight the hazardous effects of mining activities on soil, plants, and animals. HM concentrations were higher in the soil and *L. shawii* from the mining site, which demonstrates the negative environmental impact of mining. *G. nanus* living at the mining site exhibit hepato- and nephrotoxicity, and lung manifestations. The deleterious effects of mining activities on *G. nanus* were associated with oxidative stress and the depletion of antioxidants. These results demonstrate the environmental and health impacts of mining and can provide a scientific basis for evaluating the effects of mining on nearby communities.
